# Real-space Wigner-Seitz Cells Imaging of Potassium on Graphite via Elastic Atomic Manipulation

**DOI:** 10.1038/srep08276

**Published:** 2015-02-05

**Authors:** Feng Yin, Pekka Koskinen, Sampo Kulju, Jaakko Akola, Richard E. Palmer

**Affiliations:** 1 Nanoscale Physics Research Laboratory, School of Physics and Astronomy, University of Birmingham, Edgbaston, Birmingham, B15 2TT, UK; 2 School of Physics and Information Technology, Shaanxi Normal University, Xi'an 710062, PR China; 3 Nanoscience Center, Department of Physics, FI-40014 University of Jyvaskyla, Finland; 4 Department of Physics, Tampere University of Technology, FI-33101 Tampere, Finland; 5 COMP Centre of Excellence, Department of Applied Physics, Aalto University, FI-00076 Aalto, Finland

## Abstract

Atomic manipulation in the scanning tunnelling microscopy, conventionally a tool to build nanostructures one atom at a time, is here employed to enable the atomic-scale imaging of a model low-dimensional system. Specifically, we use low-temperature STM to investigate an ultra thin film (4 atomic layers) of potassium created by epitaxial growth on a graphite substrate. The STM images display an unexpected honeycomb feature, which corresponds to a real-space visualization of the Wigner-Seitz cells of the close-packed surface K atoms. Density functional simulations indicate that this behaviour arises from the elastic, tip-induced vertical manipulation of potassium atoms during imaging, i.e. elastic atomic manipulation, and reflects the ultrasoft properties of the surface under strain. The method may be generally applicable to other soft e.g. molecular or biomolecular systems.

Atomic manipulation by the STM tip is generally accompanied by ‘passive' STM imaging (e.g. at lower current) to reveal the arrangement of atoms before and after the manipulation event(s)^1^,^2^,^3^,^4^,^5^,^6^,^7^,^8^,^9^,^10^,^11^,^12^,^13^. In some circumstances, however, the process of ‘passive' STM imaging itself perturbs the electrons and even the atoms themselves in the surface system. The interaction between the STM tip and the sample can enhance the corrugation of the surface and induce a rich variety of STM image patterns in the experiments^14^,^15^,^16^,^17^,^18^,^19^,^20^,^21^,^22^,^23^. In this letter, we demonstrate that the perturbation of STM tip can be utilized to obtain real-space Wigner-Seitz cell images of the surface atoms in ‘supersoft' systems (K/graphite). At the fundamental level, this “elastic atomic manipulation” arises from the ‘supersoft' nature of the epitaxial K layer as a result of the substantial tensile strain. The behaviour we observe is most unexpected.

The K/graphite system is of interest because potassium shows intriguing structural phase transitions on graphite at temperatures below 90 K, exhibiting rich phase behaviour as the adatom coverage is increased^19^,^24^,^25^,^26^,^27^,^28^,^29^,^30^,^31^,^32^,^33^,^34^,^35^,^36^,^37^,^38^,^39^,^40^. Above a critical K density a close-packed (2 × 2) phase starts to form. The lateral K-K atomic spacing in this structure is 4.92 Å, considerably larger than the atomic spacing in bulk BCC K (4.57 Å)^41^. Thus K/graphite is an ideal system for studying the effects of tensile strain on a “simple metal”. STM is a natural tool to image the atomic structures of the system in the sub-monolayer regime, where the electronic contrast between adatoms and support is considerable. However, as the K film thickness increases into the multilayer regime, we would expect to lose atomic detail in the images. After all, K is a classical free electron metal.

## Results

**Figure 1:**
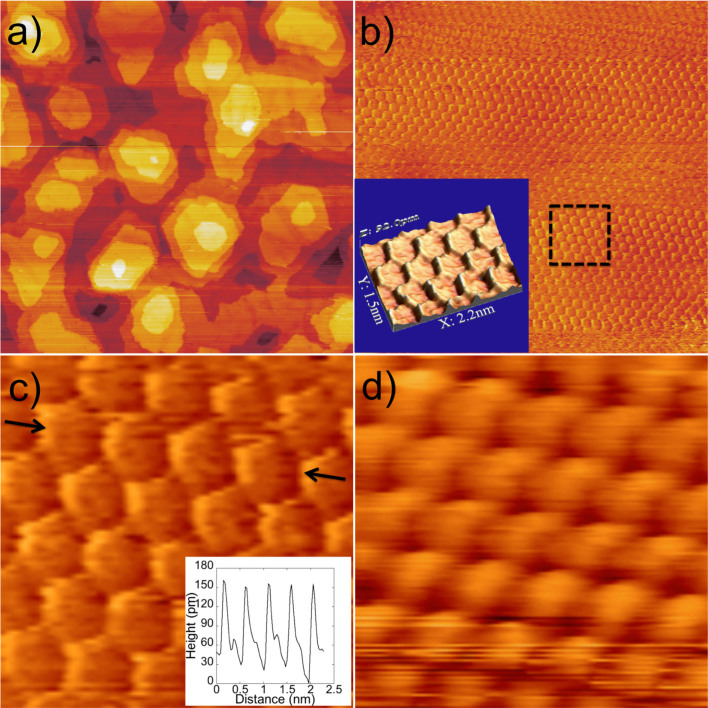
Constant current LT-STM images of K/graphite. (a) Constant current STM (at -2.0 V, 20 pA) image (300 nm × 300 nm) of K multilayer film on graphite. (b) Atomic resolution STM (at −1.5 V, 30 pA) image (15.7 nm × 15.7 nm) of a fourth layer terrace; inset a three-dimensional visualization of the part of STM image (2.2 nm × 1.5 nm) showing the Wigner-Seitz cell. (c) Zoomed in image of the area marked by the dashed square (2.5 nm × 2.5 nm) in panel (b); inset, the line profile along the line pointed to by the arrows. (d) Atomic resolution STM (at −1.6 V, 100 pA) image (2.5 nm × 2.5 nm) of a fourth layer terrace when the tip condition changed after repeated positive bias scans (+2.0 V, 10 pA). The scanning direction of all images was horizontal from left to right.

**Figure 2:**
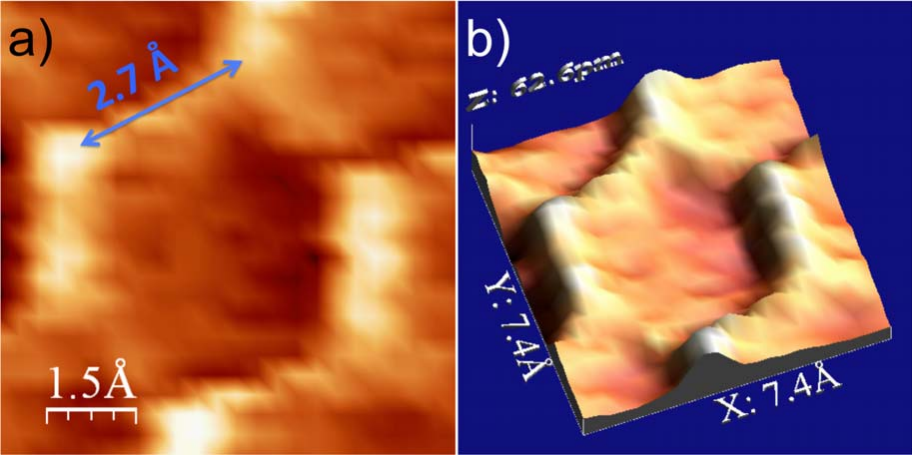
The honeycomb feature of K film. (a) Zoom in STM image of a single honeycomb STM (at −1.5 V, 30 pA). (b) Three-dimensional visualization of the STM image in (a).

The STM topography of the K multilayer surface on graphite at 4.4 K (sample bias voltage -2.0 V, current 20 pA) shows good layer-by-layer growth (Fig. 1a). The first two layers cover almost the entire surface and the third layer is still continuous, but further layers display island structures with random shapes. The layers are atomically flat, consistent with a replication of the close-packed (2 × 2) phase found in the saturated monolayer, the subject of several previous experimental and theoretical investigations at temperatures below 90 K^19^,^24^,^25^,^26^,^27^,^28^,^29^,^30^,^31^,^32^,^33^,^34^,^35^,^36^,^38^,^39^,^40^. However, zooming in on an island of height four atomic layers with atomic resolution reveals an unexpected honeycomb network structure (Fig. 1b). This structure, which is not observed on films of 1–3 monolayers high, structure is emphasized in high-resolution STM images (Fig. S1), which show the side edges of the hexagons to be considerably higher (about 90pm) than their centres (Figs. 1c and 2). Quantitative analysis yields a side length of 2.7 Å (Fig. 2b), markedly smaller than the nearest-neighbour spacing in bulk K. The lattice constant of the honeycomb is about 5 Å, close to the K-K atom spacing of 4.92 Å in the close-packed (2 × 2) monolayer phase on graphite. However, the honeycomb pattern cannot be a reflection of the graphite support beneath, because the four-layer film is too thick and the dimensions of the network are in any case a factor two too large. Critically, giving rise to the honeycomb structure, the symmetry of the close-packed surface and the distances measured imply that the honeycomb K atoms reside at the *centers* of the hexagons. This in turn means that the close-packed surface gives STM heights which are, counter-intuitively, larger at bridge and hollow sites, and smaller at top sites. Therefore, the honeycomb network is a direct visualization of the Wigner-Seitz cells of the surface atoms.

The honeycomb feature is impossible to understand via a picture of static electronic and atomic structures. The sides of the honeycomb perpendicular to the scanning direction (horizontal in Fig. 2a) are brighter than the sides at 30° to it. Since the scanning direction affects the observations, the honeycomb cannot arise from the intrinsic electronic structure. The fine details observed also depend on surface bias and tip condition. Using positive surface bias the honeycomb feature disappears (Fig. S2). It also disappears (Fig. 1d) when the tip condition changes during repeated positive bias scans (+2.0 V, 10 pA). A change in the tip condition renders the hexagon edges darker and the centers brighter, but the dimensions and the symmetry remained (Fig. 1d).

The observations therefore force us to address some rather puzzling questions: What is the origin of the honeycomb pattern? If the feature is explained by tip-induced changes in the electronic structure, how can the scanning direction affect the observations? Does the tensile strain of the film produce the behaviour observed?

**Figure 3:**
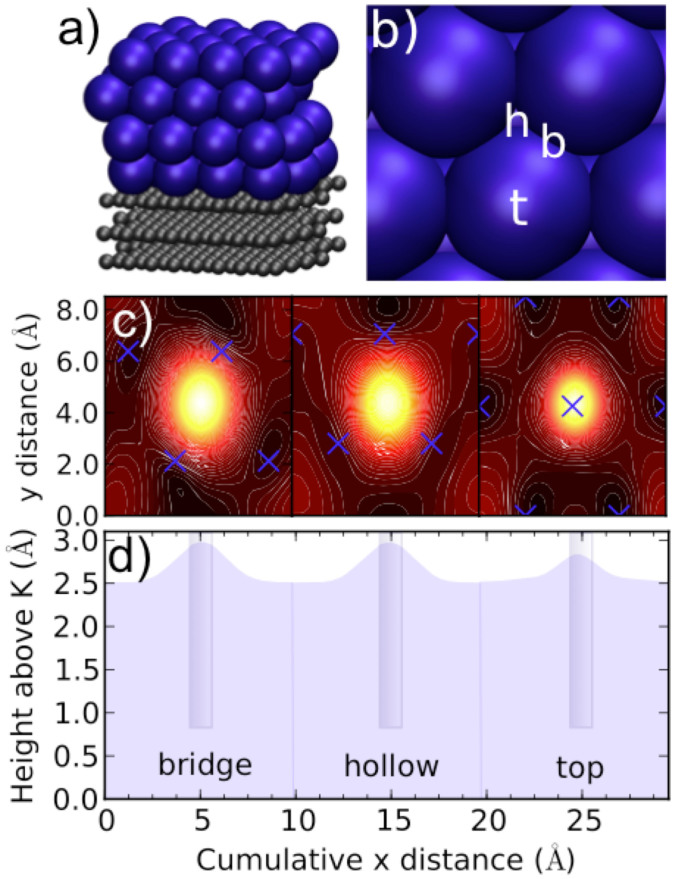
Local electronic compressibility. (a) Model system, 4 monolayers of K on 3 layers of graphite. (b) The close-packed K surface with bridge (b), hollow (h), and top (t) sites. (c) Constant electron density contours (1.4 × 10^−3^ Å^−3^) with the electron-attracting pipet at bridge (left), hollow (middle) and top (right) sites. (d) Line scan across the three labeled points in panel b, showing the same constant electron density surface. Cylinders illustrate the geometry of the electron-attracting pipet, as they are immersed into the sea of electrons.

To get theoretical insight into the honeycomb observations, a model system of four close-packed K monolayers (fcc, ABCA stacking) on top of a three-layer graphite support was simulated using the density functional theory (DFT), and the real-space grid code GPAW^42^,^43^. The simulation system contained 32 carbon atoms and 4 potassium atoms per layer, 10 Å vacuum on top of the K film, and no periodic boundary conditions in the vertical direction (lateral dimensions 8.53 Å × 9.84 Å). As suggested by previous experiments and theory^19^,^36^, K atoms were located at the centers of the graphite hexagons to form a close-packed surface (Fig. 3a). This geometry creates 10% tensile lateral strain for K as compared with the bulk. The average binding energy per K atom is 0.903 eV/atom, and an additional calculation for ABAB stacking (hcp) gives the same result (within this accuracy), also in terms of the potassium film thickness (14.3 Å) and effective charges of the surface atoms (-0.04 e). Therefore, we conclude that the film stacking may vary in the experiments but it should not affect the properties of the top K layer.

Simulation of the electronic ground state of the model system revealed a free-electron, jellium-like electronic structure at the surface, as expected. The electron density (jellium) and its single-particle wave functions near the Fermi-level did not show any honeycomb features. Moreover, a laterally homogeneous external field, such as an electric field perpendicular to the surface^19^, was also unable to induce such features. The smoothness of the jellium at the surface also implies that the honeycomb features cannot arise from tunnelling orbital effects. In particular, if the features arose from the electronic structure exclusively, the STM images would be unaffected by the scanning direction because of the swiftness of the electron dynamics. The above observations thus suggest that the imaging process must involve a dynamical movement of the atoms.

To investigate the prospect of surface atom movements initiated by the STM tip, we calculated the energies required to displace surface K atoms. Displacements up to 0.3 Å in amplitude in both lateral and vertical directions cost only 10 meV/atom, demonstrating an exceptionally shallow potential energy landscape. For comparison, this is some 400 times shallower than for a corresponding C displacement in graphite. The shallowness is due to the tensile strain in the K/graphite system, as a reference calculation for normal K bulk distances gives a 20% larger vertical confinement energy and a lateral confinement twice as large. Thus, the underlying graphite support, by creating tensile strain in K, creates an exceptionally shallow (“ultrasoft”) potential energy landscape for the surface atoms, enabling their enhanced movement in response to the tip.

However, a tip-induced atomic displacement would require the jellium's electronic response to be laterally inhomogeneous, since the movement of atoms is of course a response to the electronic density. In order to understand the response of the electron density in the presence of the tip, we immersed a cylinder-shaped potential well, or “pipet,” into the jellium. The pipet had a diameter of 1.0 Å, well depth of -10 eV, and bottom 0.8 Å above the plane of the K nuclei. We positioned it on bridge, hollow and top sites using fixed atomic geometries (Fig. 3c). The pipet, operating like a capillary sucking up liquid, attracted electrons upwards, more on bridge and hollow sites, and less on top sites (Figs. 3c and 3d). This trend indicates that *the local electronic* *compressibility on top sites is smaller*, which is plausible due to the immediate vicinity of the K ions. Smaller electronic compressibility consequently hints towards a reduced local atomic response at top sites.

To build on this insight and unify the theoretical picture, we investigated surface atom movement using an explicit model for the STM tip. While the experiments used a Pt/Ir tip, the precise condition of the tip apex is unknown, as is typical for STM measurements. Therefore, to simulate the electric field due to the applied bias voltage, we chose a positively charged apex of an ionic tip structure. We placed the tip on a bridge site, initially at an elevation of 4.0 Å above the K atoms, and relaxed the K layers below. This process was repeated for hollow and top sites. The tip elevation was large enough to mimick an STM tip at tunneling distance range, and to make the detailed choice of the tip unimportant, but small enough to induce small, perturbative movement of the surface atoms. The approach is rather coarse, but explicit constant-current STM simulations with self-consistent and concerted atom relaxation together with tip movement would not have been feasible in this context. The approach explored proved to be reasonable, because electronic structure analysis showed that the modifications to the jellium due to the presence of the tip were indeed gentle; the displaced atoms and the surrounding jellium followed each other's movements smoothly. This elastic atomic manipulation by the tip directly generates changes in the STM heights.

**Figure 4:**
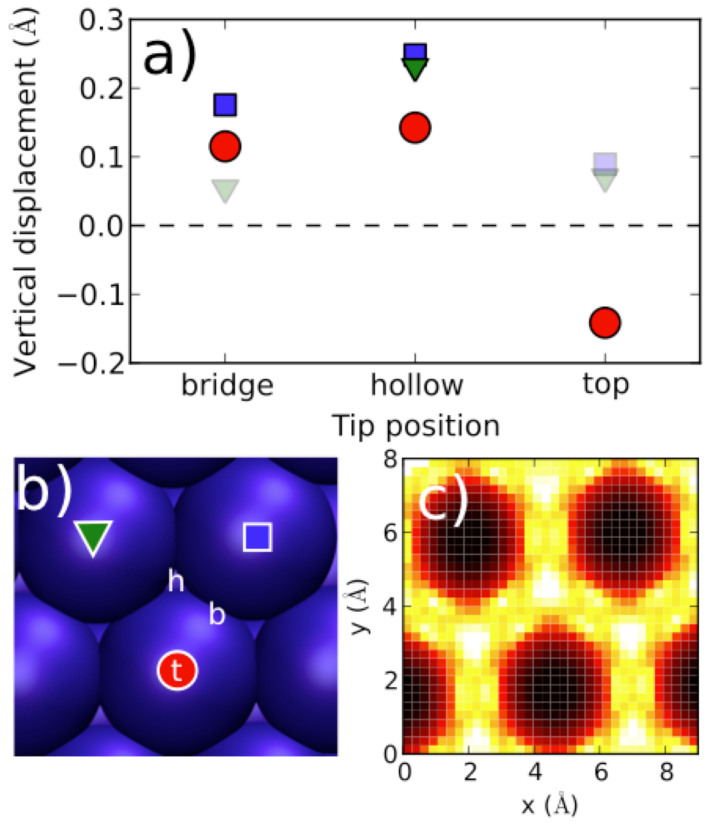
Atom relaxation with an explicit tip model. (a) Vertical displacements of the three atoms surrounding the tip (as marked in panel b). (b) Different sites for the tip (bridge, b; hollow, h; top, t) and notations for the atoms surrounding the tip (symbols). (c) Contour plot of the averaged vertical movements of the atoms surrounding the tip during a scan across the entire surface.

Simulations of the surface atom movements using this tip model showed that the direction of atomic movement depended on the lateral site (Fig. 4a). On bridge and hollow sites the tip elevated the surrounding atoms, whereas on the top site it pushed down the atom beneath. This trend demonstrates that the STM height is larger on bridge and hollow sites, and smaller on top sites. We also scanned the entire surface, moving the tip about at a constant height while quasi-statically relaxing the surface. The simulated STM height at a given location was averaged from the vertical movements of the atoms surrounding the tip by using inverse distance weighting (Fig. 4c)^44^. The resulting image is a rough representation of the STM image, and it exhibits the honeycomb pattern. We conclude that the honeycomb network arises because the electron density at top sites has smaller compressibility and weaker response, which induces weaker interaction with the tip and accordingly smaller (in fact, reverse) vertical movement of the atom; the larger electronic response in the middle of surface K-K bonds induces larger atom movement and makes the Wigner-Seitz cells visible.

To simulate positive surface bias voltage, we repeated the simulation using a tip apex with a negative local charge. Now the atoms near the tip at bridge, hollow, and top sites all move upwards together. This different trend is in qualitative agreement with the absence of the honeycomb feature in the positive surface bias experiment (Fig. S1).

## Discussion

We believe that our modeling should correctly capture the observed phenomena in the K/graphite system at a qualitative level. In experiments the tip responds dynamically to atomic movements in order to keep the current constant. Since the model uses fixed tip heights, it cannot give quantitative estimates for the STM heights; however, the trends should be correct. The experiment also shows that the hexagon edges are asymmetric and depend on scanning direction. This asymmetry can be understood to result from a hysteretic process, due to the coincident time scales for the motion of the tip and the surface atoms; our model with its quasi-static relaxation cannot account for this effect. Overall, the electronic compressibility, shallow energy landscape, and the qualitative response to an explicit tip in the simulations provide a coherent physical picture in phenomenological agreement with the experimental observations.

Finally, let us consider the specific requirements to observe the new phenomenon identified here. Firstly, it is necessary to have a jellium-like metallic surface, since covalent bonds are too rigid for such a large displacement to be induced by the weak interaction with a tip in the tunneling regime. Secondly, a sharp STM tip is needed to avoid averaging the features by the bluntness of the tip. Thirdly, this sharpness is relative to the surface bond distances. Covalent radii comparable to K occur only in Rb and Cs. We have noted that the bond distances are enlarged by the tensile strain in the K/graphite system. Fourthly, the material has to be soft (as is true of the larger alkalis): the surface atoms have to be confined weakly enough for the STM tip to induce atom movement even under a weak interaction. The bulk modulus of potassium (3.1 GPa) is among the smallest in the periodic table, and one can easily cut potassium with a knife (Rb and Cs are even softer). Moreover the tensile strain and fcc packing created by the graphite substrate decrease the structural stability of the K multi-layer film. These requirements do not mean that the phenomenon would be unique. Rather, by understanding them we can understand, or design the conditions to observe the phenomenon using other materials or settings when “elastic atomic manipulation” can be employed in other systems. Weakly bound molecular systems or biological systems may be a case in point.

In conclusion, the application of tensile stress to a soft metal such as potassium makes it “ultrasoft” - both electronically and structurally. The response of the electron density for a 4 multi-layer film of K/graphite varies between different sites, so that tiny perturbations by an STM tip induce significant, site dependent atomic displacements. The result is the enabled real-space imaging of the honeycomb pattern representing the Wigner-Seitz cells of the surface atoms. The scanning direction of the STM tip adds an additional effect of hysteresis to the images, which can be explained in terms of structural relaxation. The phenomena observed here provide a fundamental new understanding of simple metals under extreme conditions, of the behaviour of surfaces close to reconstruction instability, and the effects of the STM tip upon the atomic structure and response of an “ultrasoft” surface system. It seems to us that there may be much potential to exploit this “elastic atomic manipulation” in future studies of molecular systems.

## Methods

### Sample preparation and STM manipulation/image

The graphite (HOPG) substrates were cleaved with tape just before loading into the ultra high vacuum (UHV) preparation chamber (base pressure of 1.3 × 10^−10^ mbar) and then annealed for 30 minutes at 450°C to obtain clean, atomically flat terraces. The potassium was evaporated onto the surface by dosing with a commercial getter (SAES Getters) with the graphite surface cooled down to 90K. Finally, the sample was transferred into the Omicron LT-STM imaging chamber with a base pressure of 1.1 × 10^−11^ mbar. The STM imaging was conducted at 4.4 K with a mechanically sheared Pt/Ir (90/10) tip. The STM images were analysed by WSxM software^45^.

### Density functional theory simulation

The DFT simulations were performed by using the real-space grid code GPAW with the PBE exchange-correlation functional^42^, a 2 × 2 k-point sampling and a grid spacing of 0.2 Å^43^. The simulation system comprised four close-packed K monolayers with a sequence ABCA (fcc) and three layers of graphite. The number of atoms was 32 carbon atoms and 4 potassium atoms per layer, and the system contained a 10 Å vacuum on top of the K film, and no periodic boundary conditions in the vertical direction (lateral dimensions 8.53 Å × 9.84 Å). K atoms were located at the centers of the graphite hexagons to form a close-packed surface. A local external electrostatic field induced by the STM tip was modeled with an electron-withdrawing “pipet” (diameter of 1.0 Å, well depth of −10 eV, and bottom 0.8 Å above the topmost plane of the K nuclei). Furthermore, to mimick positive surface bias, we used a proto-typically ionic, pyramidal-shaped Mg_4_O_3_ tip, for which Bader analysis gave atomic charges of +1.2e for Mg, −1.7e for O. Similarly, for the reverse bias we used a pyramidal-shaped Mg_3_O_4_ tip with negative apex charge.

## Author Contributions

F.Y. and R.E.P. initiated this work, P.K., S.K., J.A. performed the modelling and simulation, F.Y. built experimental set and performed the experiments, P.K., S.K., J.A. wrote the simulation parts of the manuscript, F.Y. and R.E.P. wrote the other parts of the manuscript. All authors reviewed the manuscript.

## Supplementary Material

Supplementary Information Supplementary information: Real-space Wigner-Seitz Cells Imaging of Potassium on Graphite via Elastic Atomic Manipulation
