# Supplementary material for: Real-space Wigner-Seitz Cells Imaging of Potassium on Graphite via Elastic Atomic Manipulation
Source: Sci Rep. 2015 Feb 5;5:8276. doi: 10.1038/srep08276 (PMC4317683; doi:10.1038/srep08276)
Supplement: Supplementary Information — Real-space Wigner-Seitz Cells Imaging of Potassium on Graphite via Elastic Atomic Manipulation [file srep08276-s1.pdf]

Supplementary information: Real-space Wigner-Seitz Cells Imaging of Potassium on Graphite via Elastic Atomic Manipulation

Feng Yin[1, 2], Pekka Koskinen[3], Sampo Kulju[4,5], Jaakko Akola[4,5], and Richard E. Palmer[1]*

[1]*Nanoscale Physics Research Laboratory, School of Physics and Astronomy, University of Birmingham, Edgbaston, Birmingham, B15 2TT, UK*

[2]*School of Physics and Information Technology, Shaanxi Normal University, Xi'an 710062, PR China*

[3]*Nanoscience Center, Department of Physics, FI-40014 University of Jyvaskyla, Finland*

[4]*Department of Physics, Tampere University of Technology, FI-33101 Tampere, Finland*

[5]*COMP Centre of Excellence, Department of Applied Physics, Aalto University, FI-00076 Aalto, Finland*

The STM image of two layered K film on graphite

Fig. S1 is the constant current STM image of two layered K film. Some "pockmarks", each surrounded by a bright ring, can be seen on the first K layer. A long-range, periodic, bright stripe pattern can be seen on second K layer. A similar structure can be observed on the third K layer. We do not observe the honeycomb feature on the 1st, 2nd and 3rd K layers.

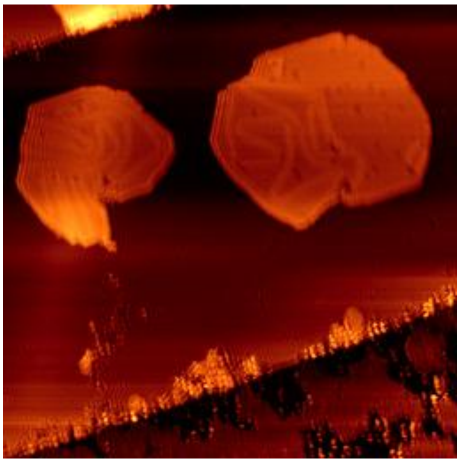

Figure S1 Constant current STM (at +2.5 V, 15 pA) image (150 nm × 150 nm) of two layer K film on graphite.

The STM image under positive bias Voltage

Fig. S2 shows the constant current STM image, which was obtained from same area as Fig. 1b at +2.0V, 10pA. From this image we can see that the honeycomb structure disappears under positive bias voltage.

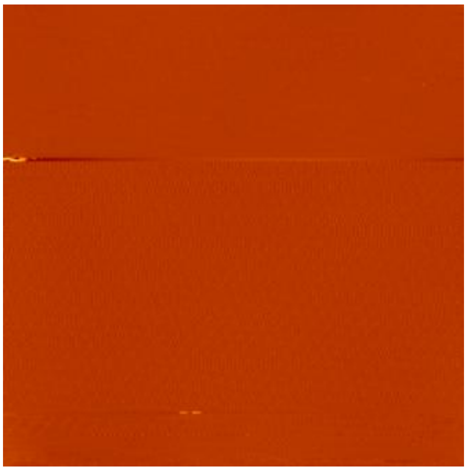

Figure S2 Constant current STM (at +2.0 V, 10 pA) image (15.7 nm × 15.7 nm) of a fourth layer terrace of multilayer film on graphite (same area as Fig. 1b).
